# Supplementary figures and images for: Transcription induces context-dependent remodeling of chromatin architecture during differentiation
Source: PLoS Biol. 2023 Dec 4;21(12):e3002424. doi: 10.1371/journal.pbio.3002424 (PMC10721200; doi:10.1371/journal.pbio.3002424)

**A**

ES Hi-C

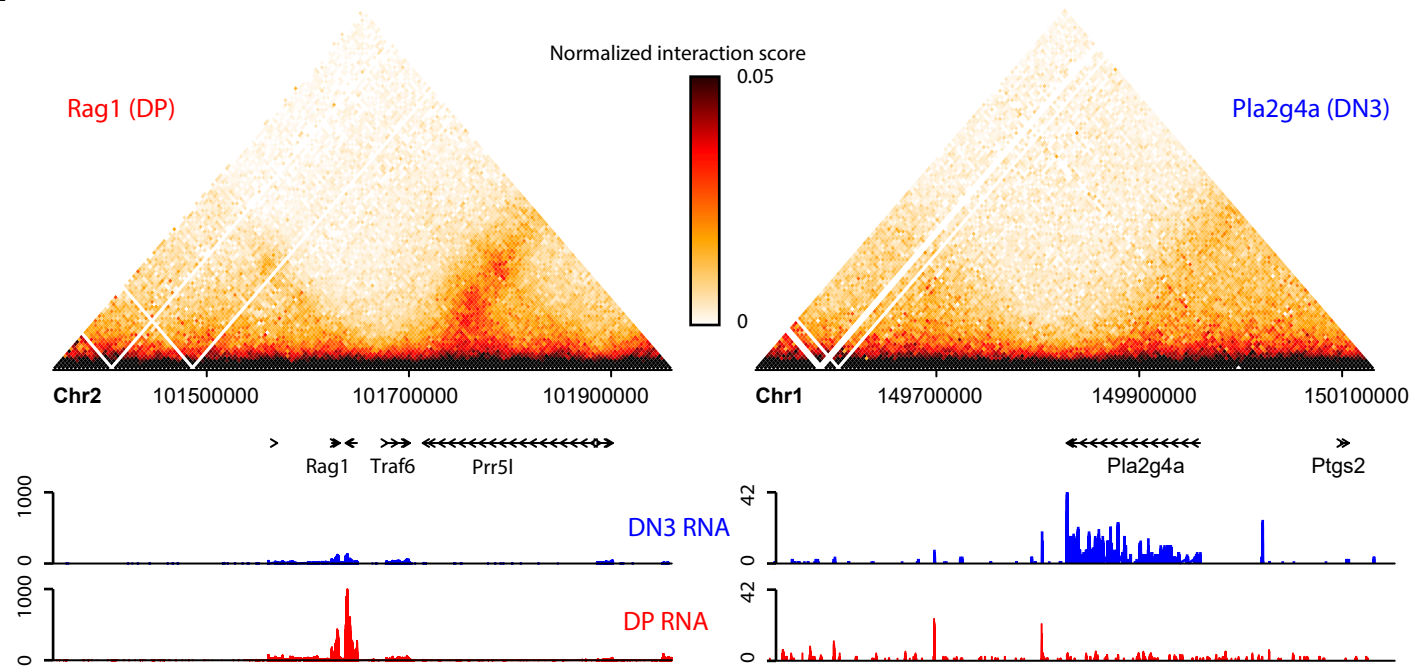
**B**
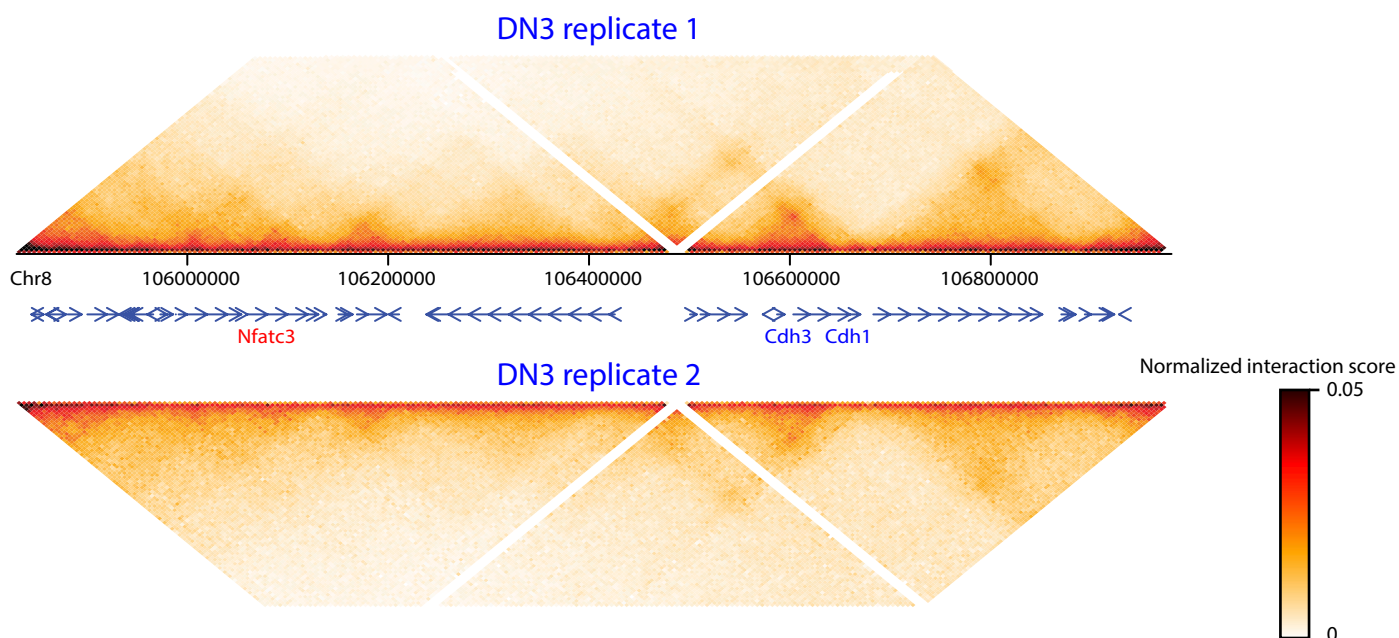
**C**
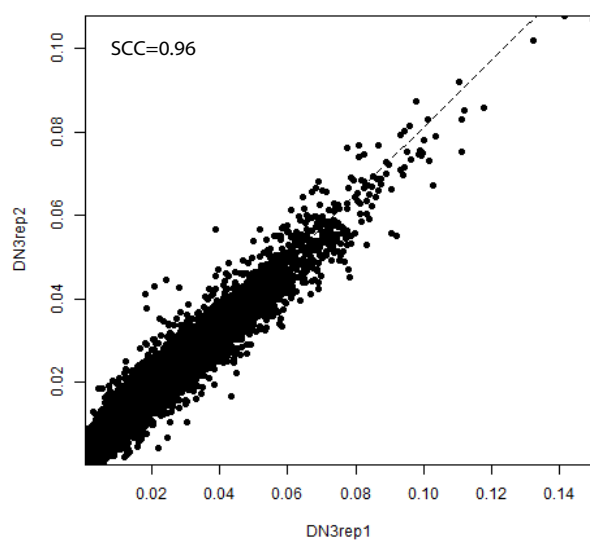
**D**
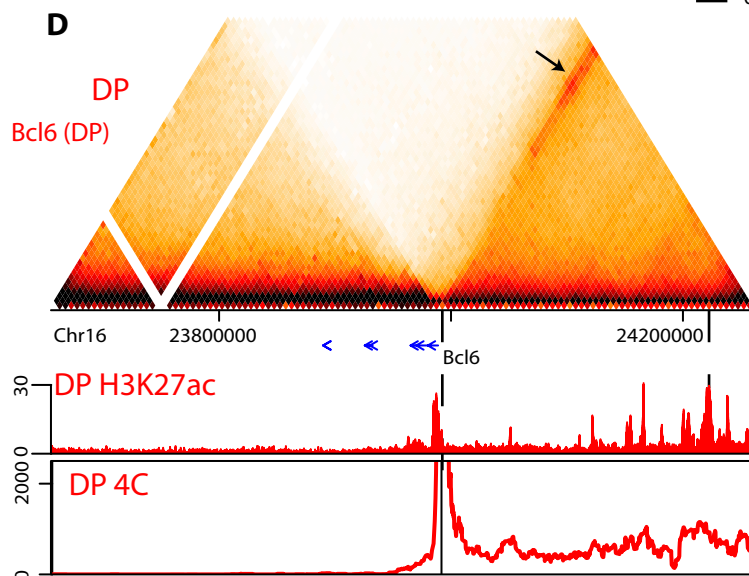

Supplement: S1 Fig — (A) ESC Hi-C maps (data taken from [21]) are shown at 5 kb resolution for approximately 600 kb regions surrounding the genes Rag1 (left) and Pla2g4a (right), showing a TAD border near these genes. Below are shown the positions of genes and RNA-seq tracks (normalized counts per million reads; non-strand-specific) from DN3 (blue) and DP (red) cells, showing differential expression of the target genes between the thymocyte populations. (B) Capture Hi-C maps for both biological replicates in DN3 cells are shown at 5 kb resolution for an approximately 1.2 Mb region, including the genes Nfatc3 and Cdh1, to show reproducibility. Positions of genes are shown. (C) Scatter plot for all normalized interaction scores from the 2 DN3 Capture Hi-C replicates. SCC is shown on the graph. (D) Pooled Capture Hi-C map for DP cells is shown at an approximately 600 kb region around the gene Bcl6. Arrow indicates a putative interaction between the Bcl6 promoter and an upstream enhancer, identified by a peak of H3K27ac in the ChIP-seq track below. 4C-seq in DP cells using the Bcl6 promoter as bait indicates sustained interactions over the broad upstream H3K27ac domain. Source data available in S1 Data. DN3, double negative; DP, double positive; ESC, embryonic stem cell; SCC, Spearman correlation coefficient; TAD, topologically associated domain. (PDF) [file pbio.3002424.s001.pdf]

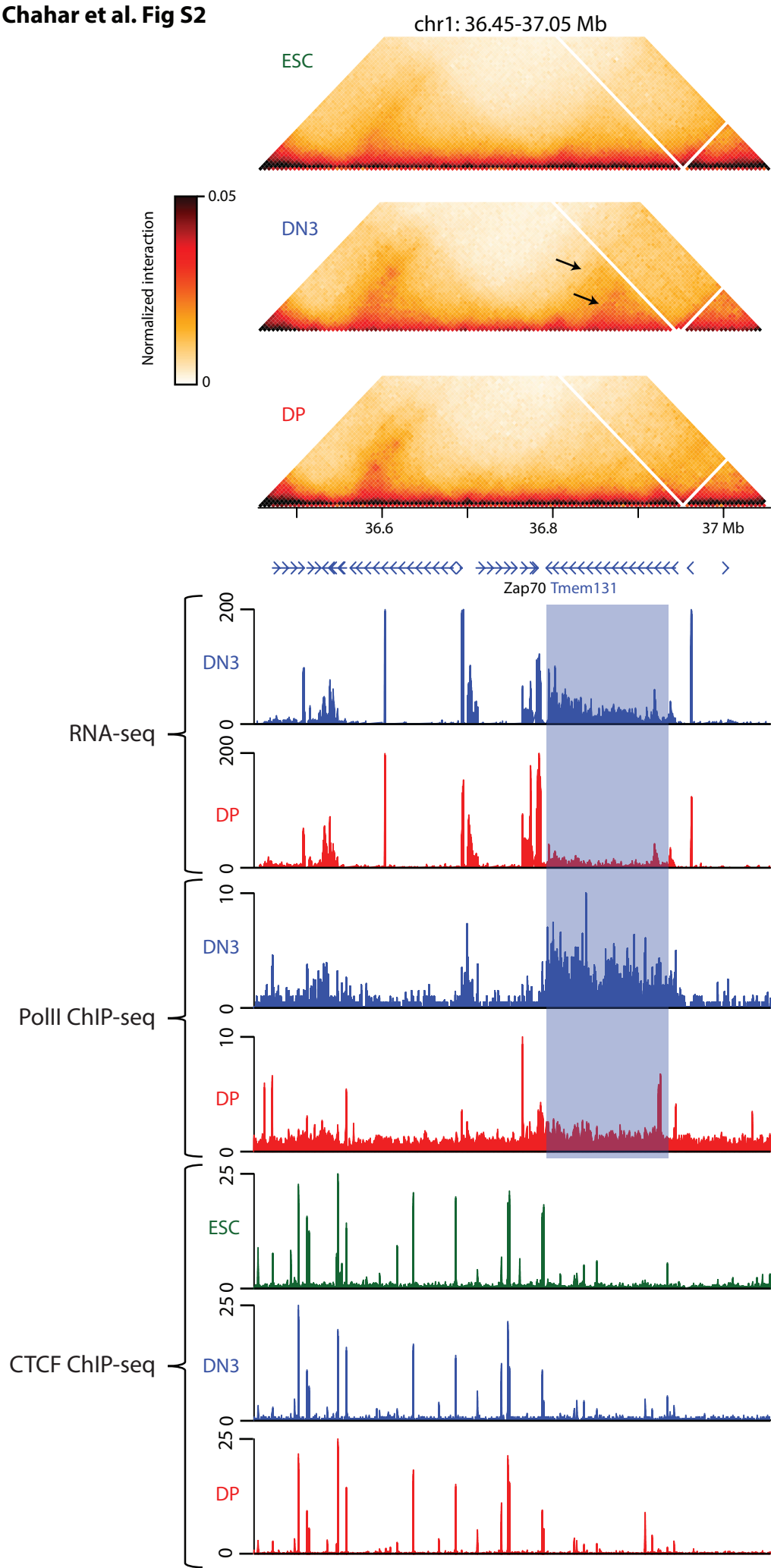

Supplement: S2 Fig — Pooled Capture Hi-C maps are shown at 5 kb resolution for an approximately 600 kb region comprising the thymocyte-expressed Zap70 and DN3-up-regulated Tmem131 genes alongside epigenomic profiles for ESCs (green), DN3 (blue), and DP (red) cells. Top to bottom: ESC, DN3, and DP Capture Hi-C maps, positions of genes, DN3, and DP RNA-seq (normalized counts per million reads; non-strand-specific), ChIP-seq (normalized counts per million reads) for RNA polymerase II and CTCF. Arrows on map and blue stripe indicates strengthened spatial domain in DN3 cells around Tmem131 gene, correlating with increased RNA polymerase binding and not associated with major changes in CTCF binding. Source data available in S1 Data. DN3, double negative; DP, double positive; ESC, embryonic stem cell. (PDF) [file pbio.3002424.s002.pdf]

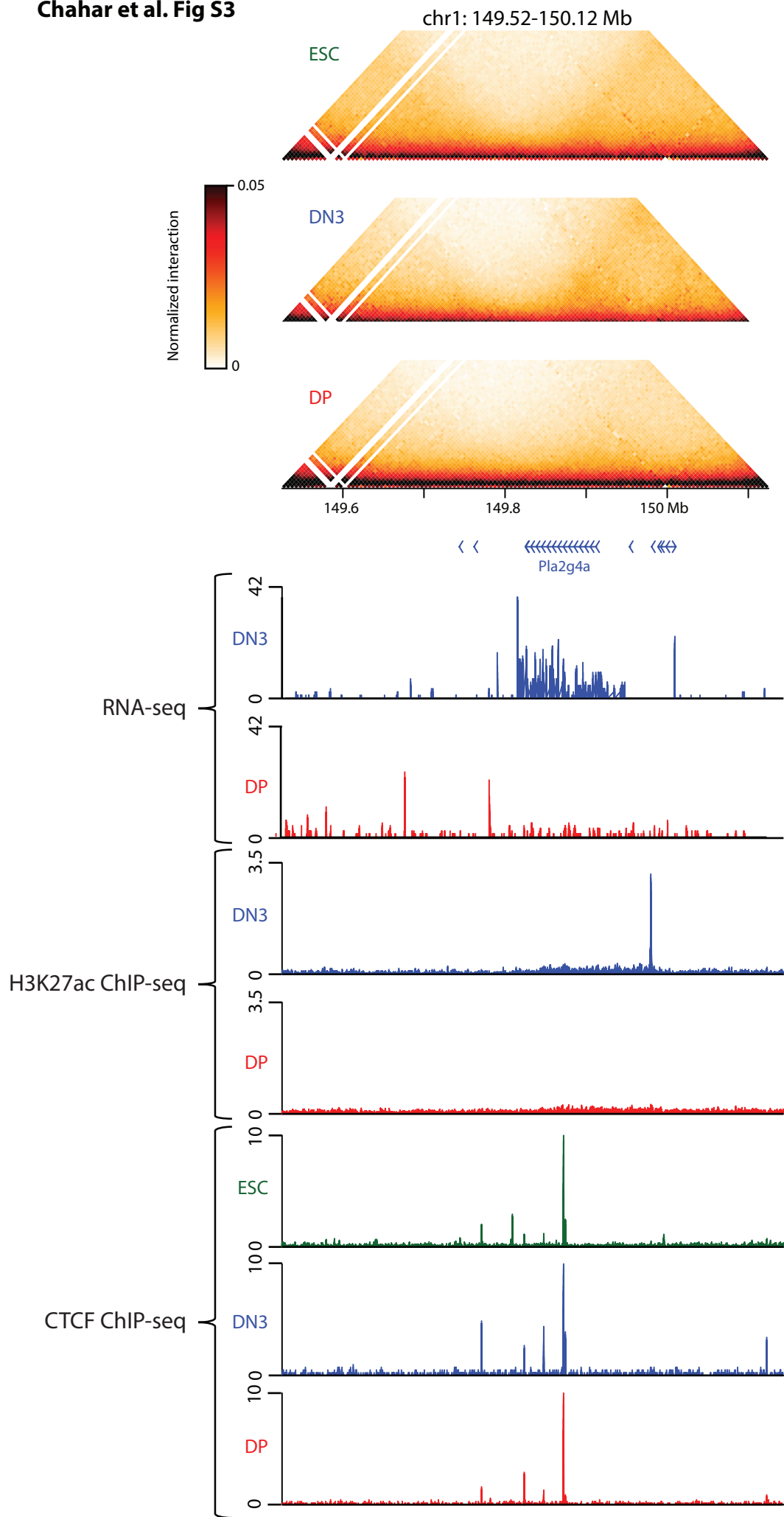

Supplement: S3 Fig — Pooled Capture Hi-C maps are shown at 5 kb resolution for an approximately 600 kb region comprising the DN3-up-regulated Pla2g4a gene alongside epigenomic profiles for ESCs (green), DN3 (blue), and DP (red) cells. Top to bottom: ESC, DN3, and DP Capture Hi-C maps, positions of genes, DN3, and DP RNA-seq (normalized counts per million reads; non-strand-specific), ChIP-seq (normalized counts per million reads) for H3K27ac and CTCF. Source data available in S1 Data. DN3, double negative; DP, double positive; ESC, embryonic stem cell. (PDF) [file pbio.3002424.s003.pdf]

chr8:105.83-106.98 Mb

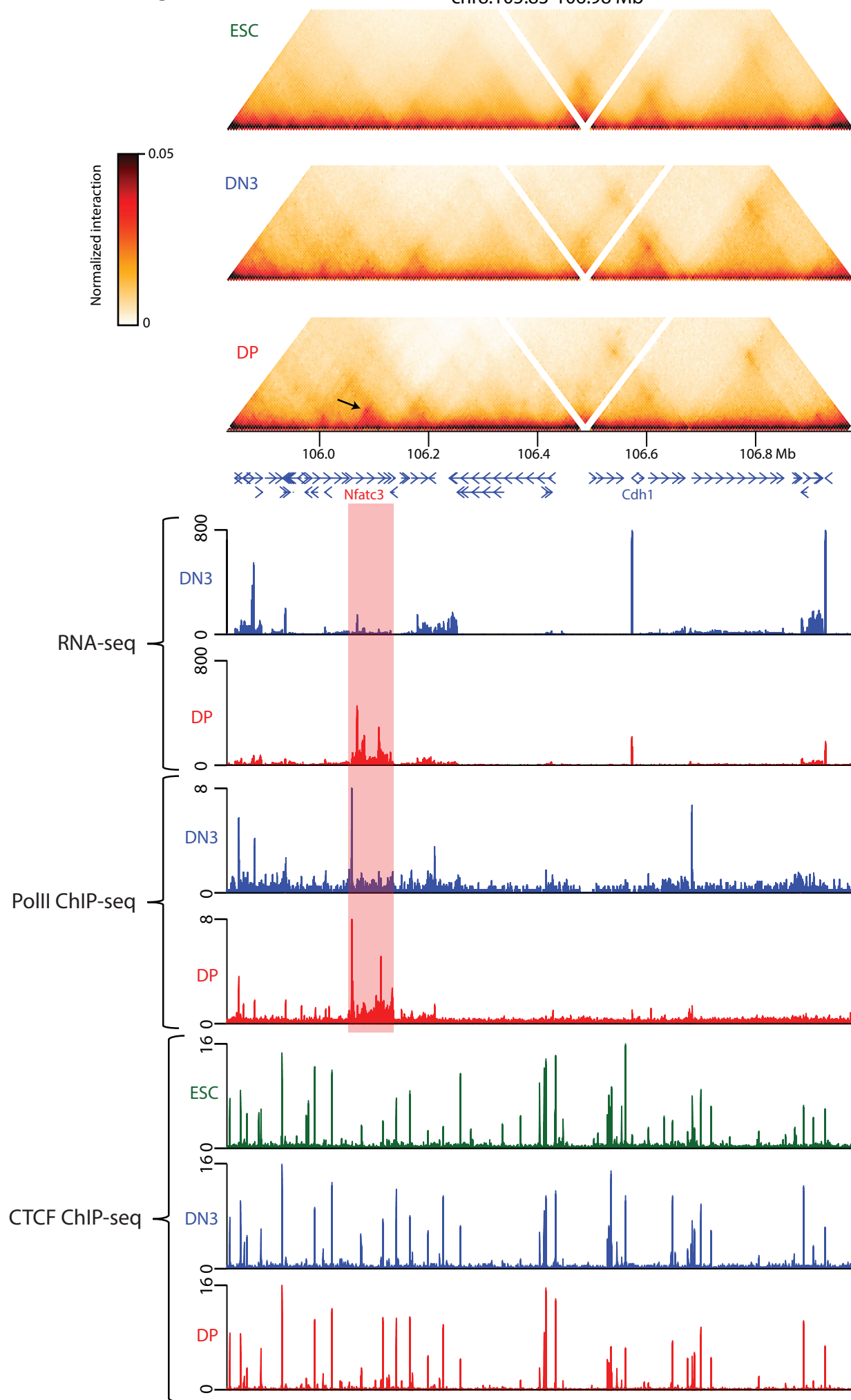

Supplement: S4 Fig — Pooled Capture Hi-C maps are shown at 5 kb resolution for an approximately 1.2 Mb region comprising the DP-up-regulated Nfatc3 and DN3-up-regulated Cdh1 genes alongside epigenomic profiles for ESCs (green), DN3 (blue), and DP (red) cells. Top to bottom: ESC, DN3, and DP Capture Hi-C maps, positions of genes, DN3, and DP RNA-seq (normalized counts per million reads; non-strand-specific), ChIP-seq (normalized counts per million reads) for RNA polymerase II and CTCF. Arrow on map and red stripe indicates strengthened spatial domain in DP cells around Nfatc3 gene, correlating with increased RNA polymerase binding and not associated with major changes in CTCF binding. Source data available in S1 Data. DN3, double negative; DP, double positive; ESC, embryonic stem cell. (PDF) [file pbio.3002424.s004.pdf]

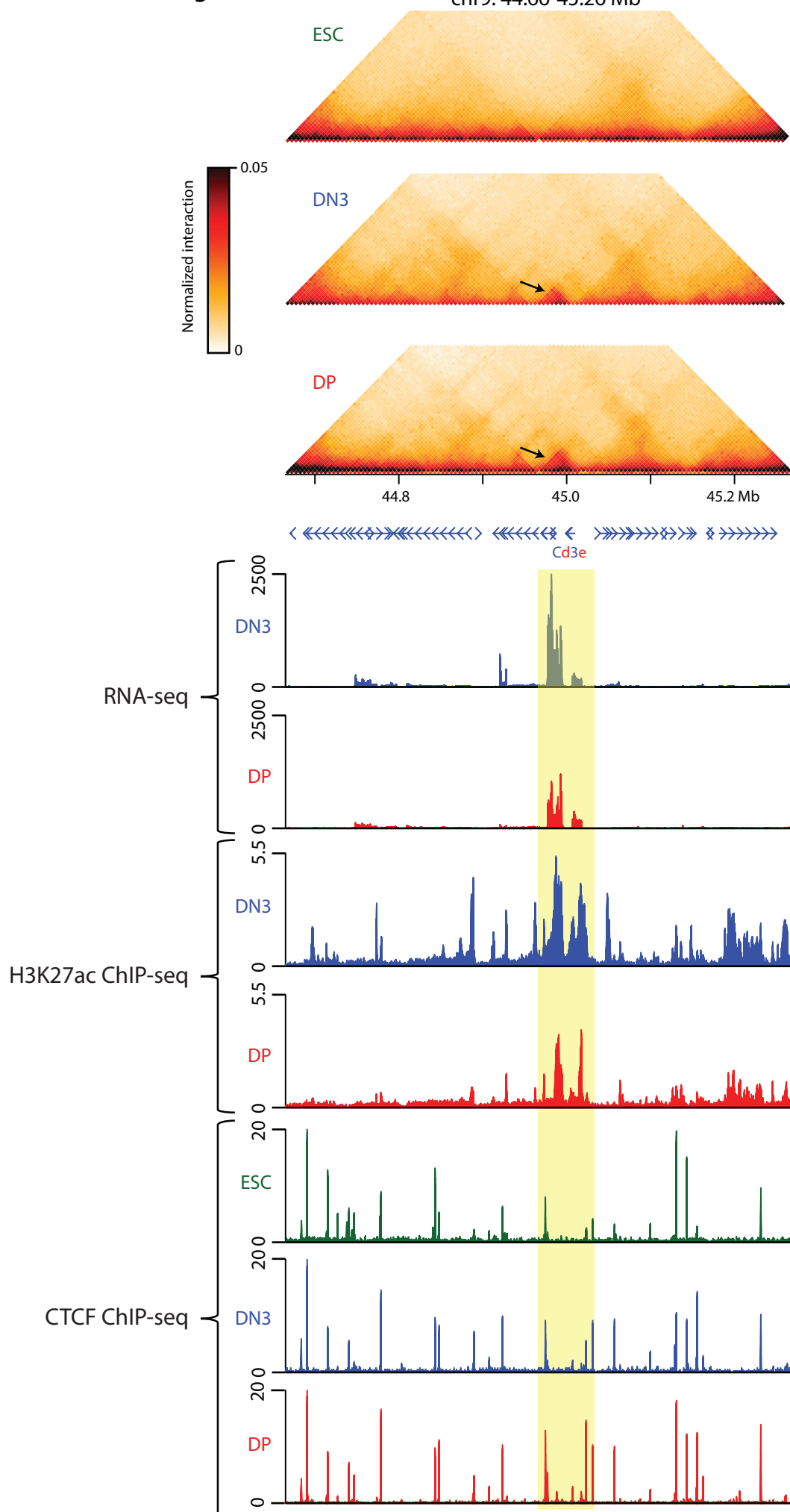

Supplement: S5 Fig — Pooled Capture Hi-C maps are shown at 5 kb resolution for an approximately 600 kb region comprising the thymocyte-expressed Cd3 gene cluster alongside epigenomic profiles for ESCs (green), DN3 (blue), and DP (red) cells. Top to bottom: ESC, DN3, and DP Capture Hi-C maps, positions of genes, DN3, and DP RNA-seq (normalized counts per million reads; non-strand-specific), ChIP-seq (normalized counts per million reads) for H3K27ac and CTCF. Arrows on maps and yellow stripe indicates strengthened spatial domain in thymocytes around Cd3, compared to ESCs, correlating with increased CTCF binding at one border. Source data available in S1 Data. DN3, double negative; DP, double positive; ESC, embryonic stem cell. (PDF) [file pbio.3002424.s005.pdf]

Chahar et al. Fig S6

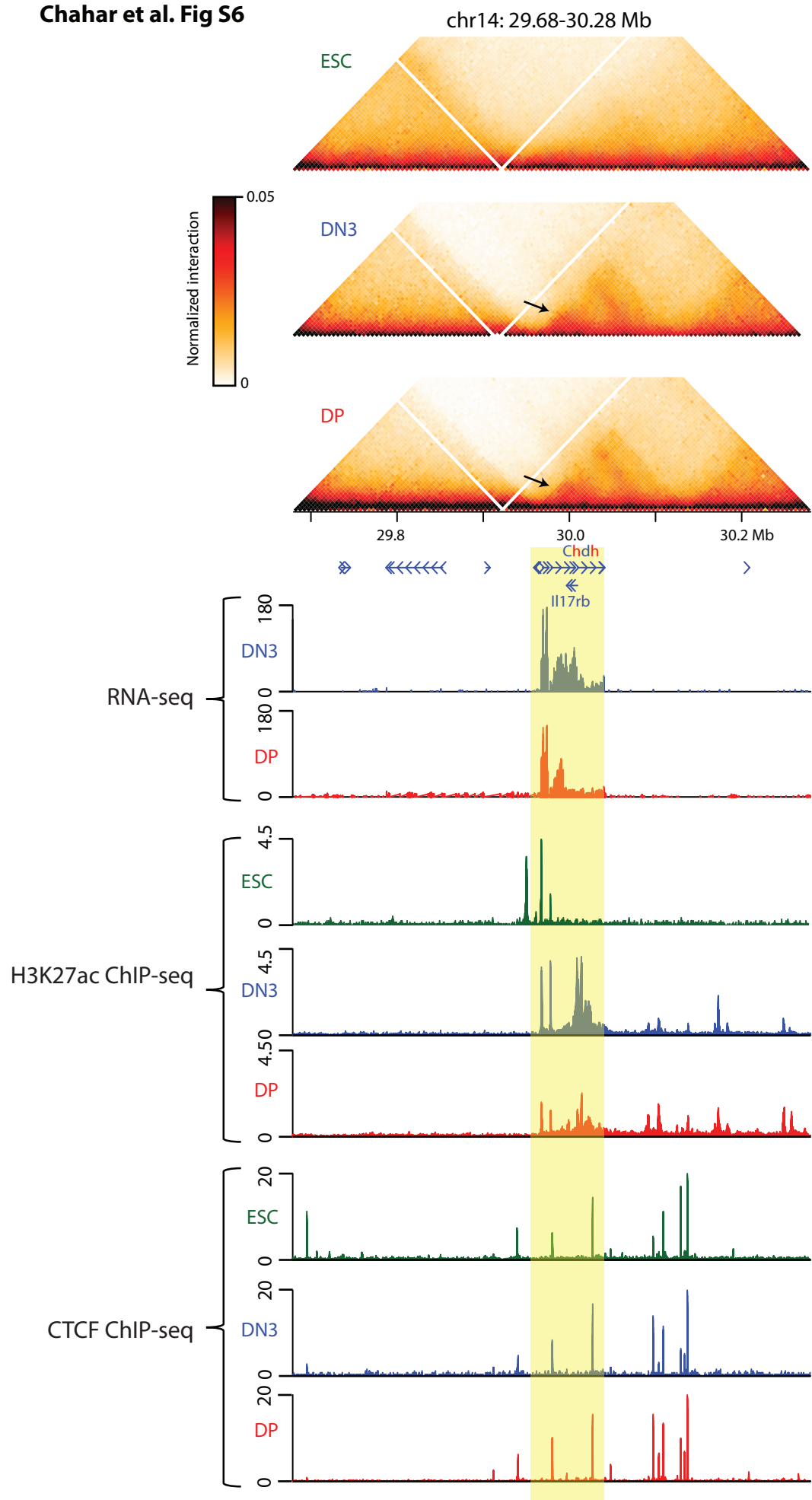

Supplement: S6 Fig — Pooled Capture Hi-C maps are shown at 5 kb resolution for an approximately 600 kb region comprising the DN3-up-regulated Il17rb gene and thymocyte-expressed Chdh gene alongside epigenomic profiles for ESCs (green), DN3 (blue), and DP (red) cells. Top to bottom: ESC, DN3, and DP Capture Hi-C maps, positions of genes, DN3, and DP RNA-seq (normalized counts per million reads; non-strand-specific), ChIP-seq (normalized counts per million reads) for H3K27ac and CTCF. Arrows on maps and yellow stripe indicates strengthened spatial domain in thymocytes around Il17rb, compared to ESCs, without apparent changes in CTCF binding at the new domain border. Source data available in S1 Data. DN3, double negative; DP, double positive; ESC, embryonic stem cell. (PDF) [file pbio.3002424.s006.pdf]

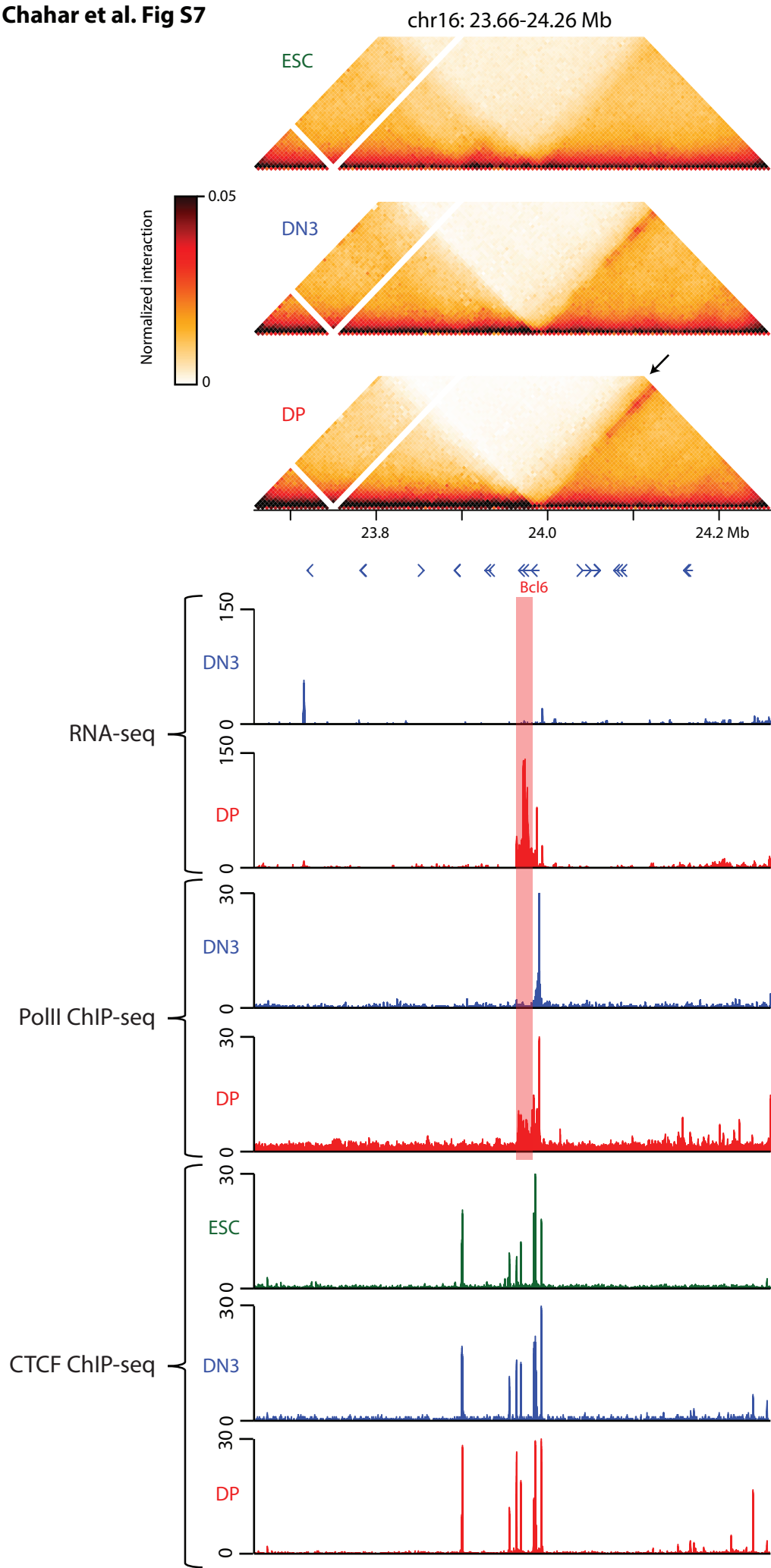

Supplement: S7 Fig — Pooled Capture Hi-C maps are shown at 5 kb resolution for an approximately 600 kb region comprising the DP-up-regulated Bcl6 gene alongside epigenomic profiles for ESCs (green), DN3 (blue), and DP (red) cells. Top to bottom: ESC, DN3, and DP Capture Hi-C maps, positions of genes, DN3, and DP RNA-seq (normalized counts per million reads; non-strand-specific), ChIP-seq (normalized counts per million reads) for RNA polymerase II and CTCF. Arrow on map and red stripe indicates DP-specific broadened TAD border, correlating with extension of RNA polymerase II into the Bcl6 gene body and accompanied by only mild CTCF binding changes. Source data available in S1 Data. DN3, double negative; DP, double positive; ESC, embryonic stem cell; TAD, topologically associated domain. (PDF) [file pbio.3002424.s007.pdf]

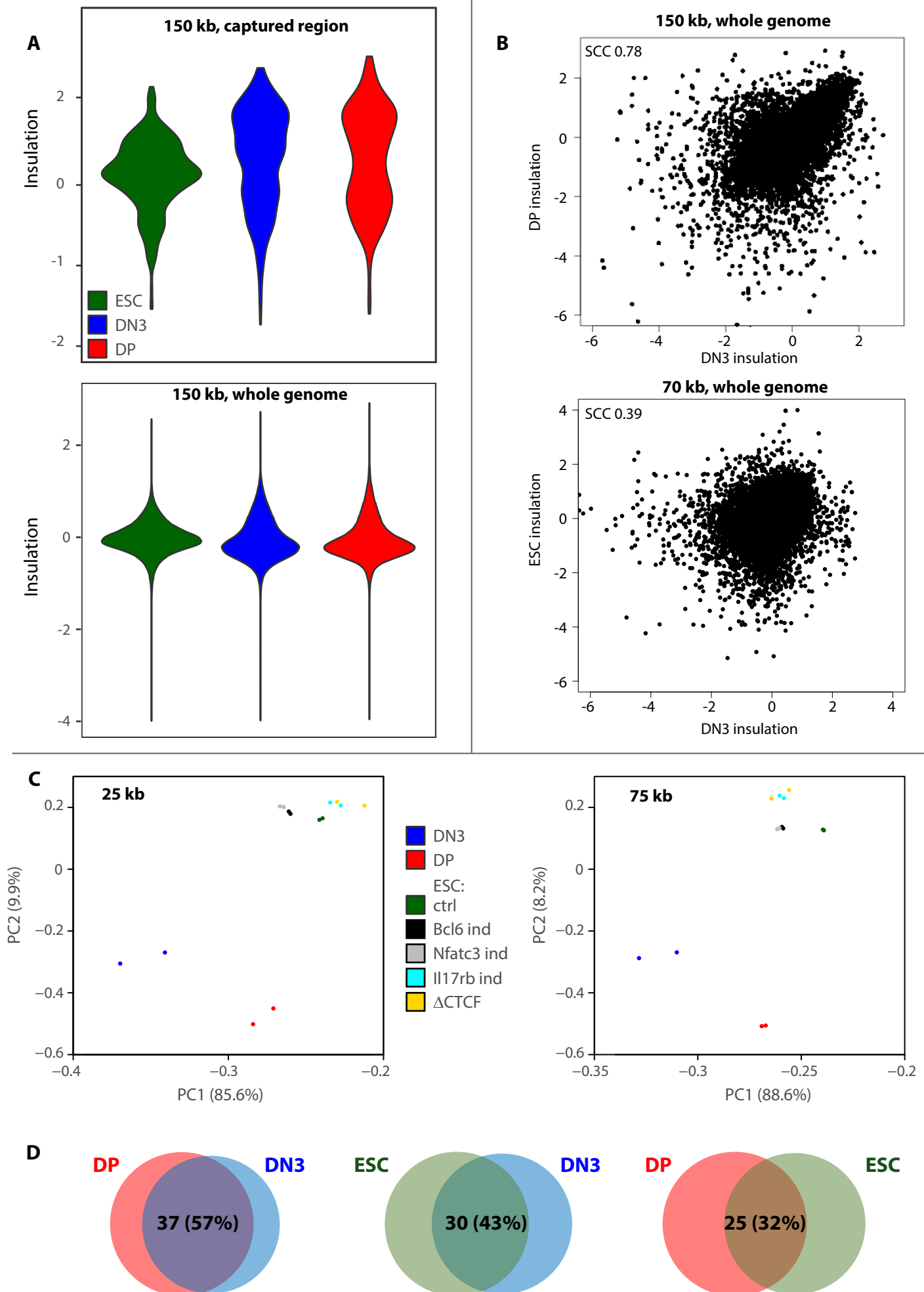

Supplement: S8 Fig — (A) Violin plots for distributions of insulation scores computed on pooled Hi-C datasets at 10 kb resolution, using a 150-kb (15 bin) window, on ESCs (green), DN3 (blue), and DP (red) cells. Whether analysis is restricted to the region targeted in the Capture Hi-C (top) or applied to the whole genome (bottom), ESCs have apparently more homogeneous insulation scores than thymocytes. (B) Scatter plots comparing insulation scores from pooled Hi-C data at 10 kb resolution. Top: comparing DN3 and DP cells using a 150-kb (15 bin) window; bottom: comparing ESCs and DP cells using a 70-kb (7 bin) window. SCC values are given on the graph. (C) Plots of first 2 principal components for insulation scores computed on biological replicates of Capture Hi-C datasets at 5 kb resolution, using a 25-kb (5 bin) window (left) or a 75-kb (15 bin) window (right) for DN3 (blue), DP (red), untreated ESCs (green), and ESCs after either homozygous deletion of the major CTCF site at the Bcl6 promoter (yellow) or CRISPRa ectopic induction of Bcl6 (black), Nfatc3 (gray), or Il17rb (cyan). (D) Pairwise Venn diagrams for overlap of called TAD boundaries from the pooled Capture Hi-C data of ESCs (green), DN3 (blue), and DP (red) cells. Source data available in S1 Data. CRISPRa, CRISPR activation; DN3, double negative; DP, double positive; ESC, embryonic stem cell; TAD, topologically associated domain. (PDF) [file pbio.3002424.s008.pdf]

Chahar et al. Fig S9

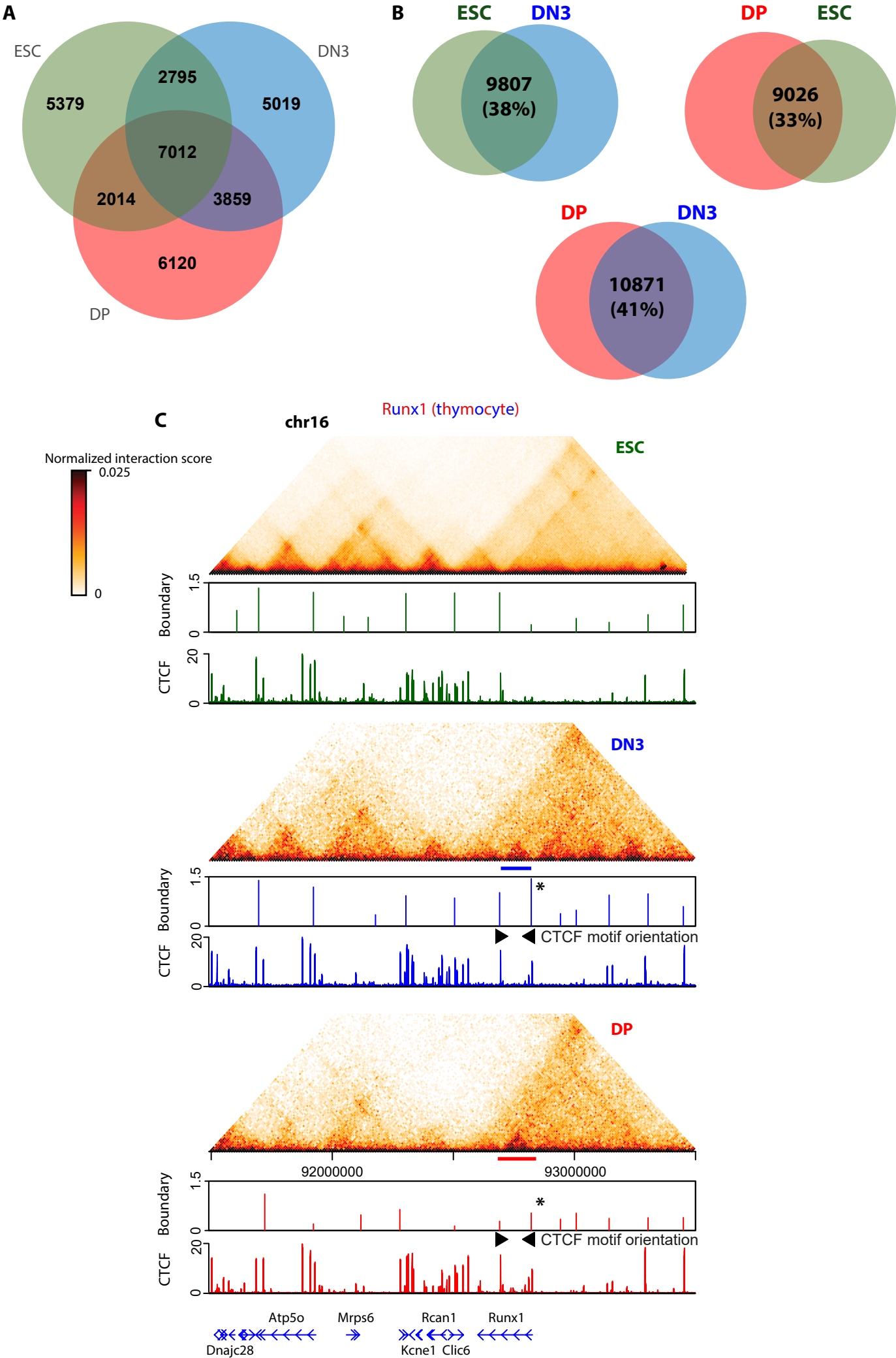

Supplement: S9 Fig — (A) Venn diagram for overlaps of called TAD boundaries from the pooled Hi-C data of ESCs (green; data taken from [21]), DN3 (blue), and DP (red) cells. (B) Pairwise Venn diagrams for the overlaps, as in (A). (C) Pooled Hi-C maps shown at 10 kb resolution for an approximately 2 Mb region around the thymocyte-expressed Runx1 gene in ESCs (top; green), DN3 (middle; blue), and DP (bottom; red) cells, just above color-coded plots showing the positions and scores of called TAD boundaries, and CTCF ChIP-seq profiles (normalized by counts per million reads). Positions of genes are shown at the bottom of the plot. Red and blue bars denote the position of a thymocyte-specific domain formed around the Runx1 gene, with a thymocyte-specific boundary corresponding to increased CTCF binding denoted by an asterisk. Triangles denote the orientation of the CTCF motifs at the border of this thymocyte-specific spatial chromatin domain. Source data available in S1 Data. DN3, double negative; DP, double positive; ESC, embryonic stem cell; TAD, topologically associated domain. (PDF) [file pbio.3002424.s009.pdf]

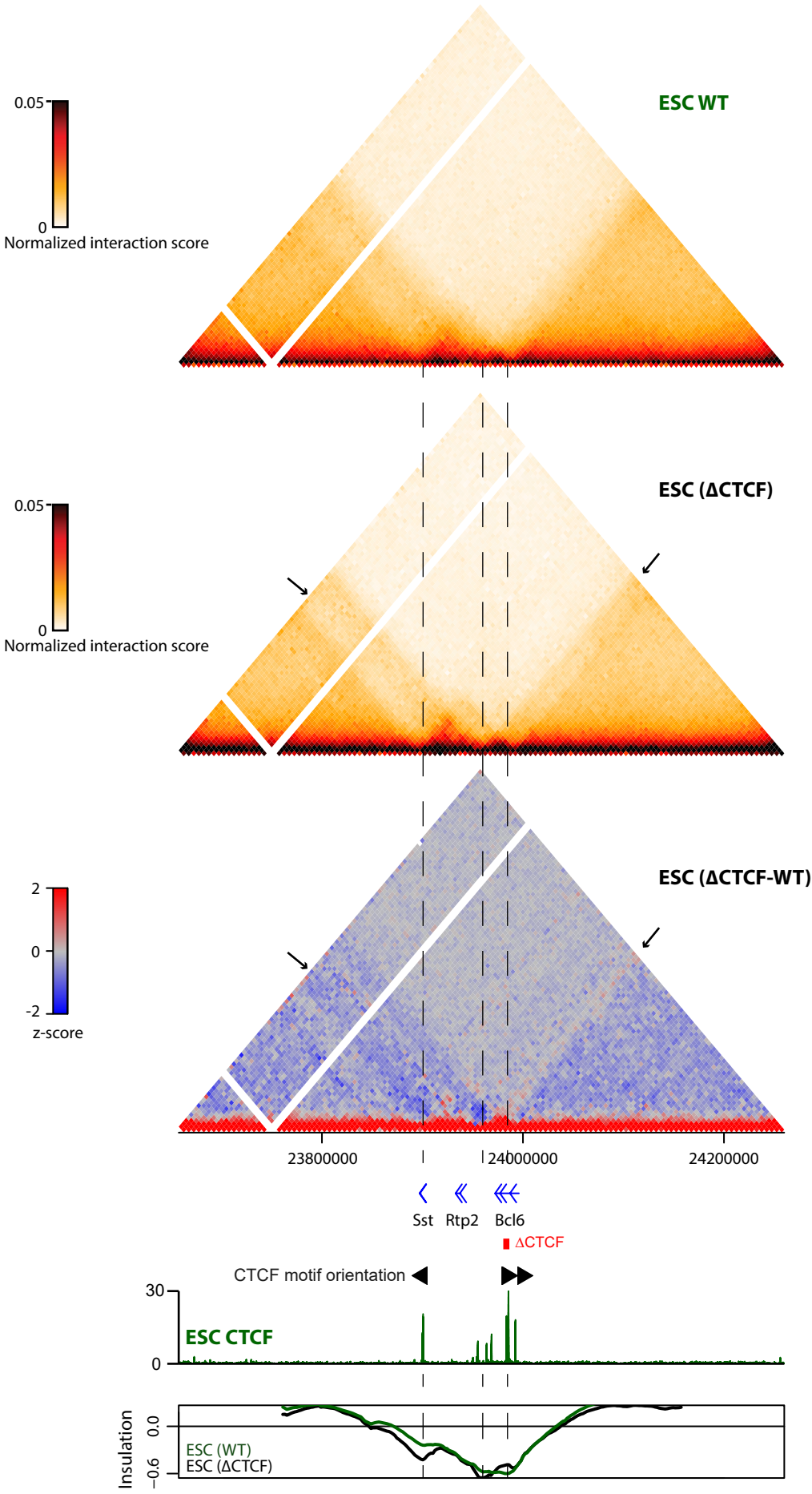

Supplement: S10 Fig — Capture Hi-C maps are shown at 5 kb resolution at an approximately 600 kb region around the Bcl6 gene in wild-type ESCs (top) and those with homozygous deletion of the major CTCF site at the Bcl6 promoter (middle), as well as the differential map (bottom) comparing the two. Below are shown the positions of genes, the position of the deletion (red), the ChIP-seq track for CTCF in ESCs, and the plot of computed insulation scores at 2 kb with a 100-kb (50 bin) window for wild-type (green) and ΔCTCF (black) ESCs. The orientations of the main CTCF motifs are indicated by arrowheads. Dashed lines show sites of altered insulation including a loss of insulation at the deleted site. Arrows on maps show where these insulation changes become apparent as stripes of relatively increased or decreased interactions. Source data available in S1 Data. (PDF) [file pbio.3002424.s010.pdf]

Chahar et al. Fig S11

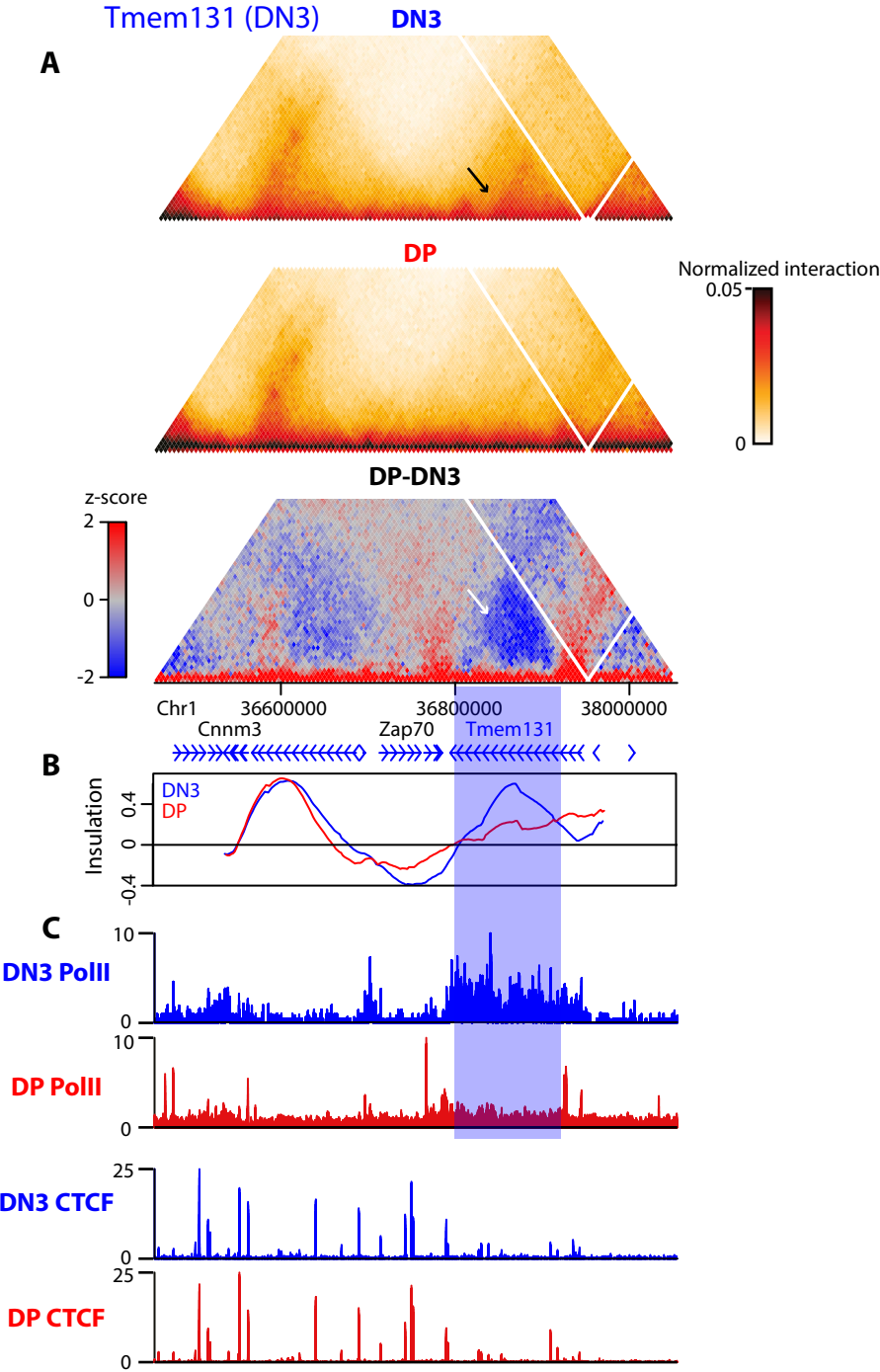

Supplement: S11 Fig — (A) Pooled Capture Hi-C interaction maps for an approximately 600 kb region around the Tmem131 gene are shown for DN3 and DP cells, above the differential heat map, where DP-enriched interactions are displayed in red and DN3-enriched interactions in blue. The arrows indicate the DN3-specific domain. Positions of genes are shown below the plots. (B) Insulation scores for the same genomic region as in (A), computed at 2 kb resolution with an 80-kb (40 bin) window for DN3 (blue) and DP (red) cells. (C) ChIP-seq profiles (normalized as counts per million reads) for the same genomic region as in (A), for DN3 (blue) and DP (red) cells. Top to bottom: RNA polymerase II, CTCF. Blue stripe indicates DN3-specific domain, with increased insulation score maximum, increased RNA polymerase II loading, and negligible changes in CTCF binding. Source data available in S1 Data. (PDF) [file pbio.3002424.s011.pdf]

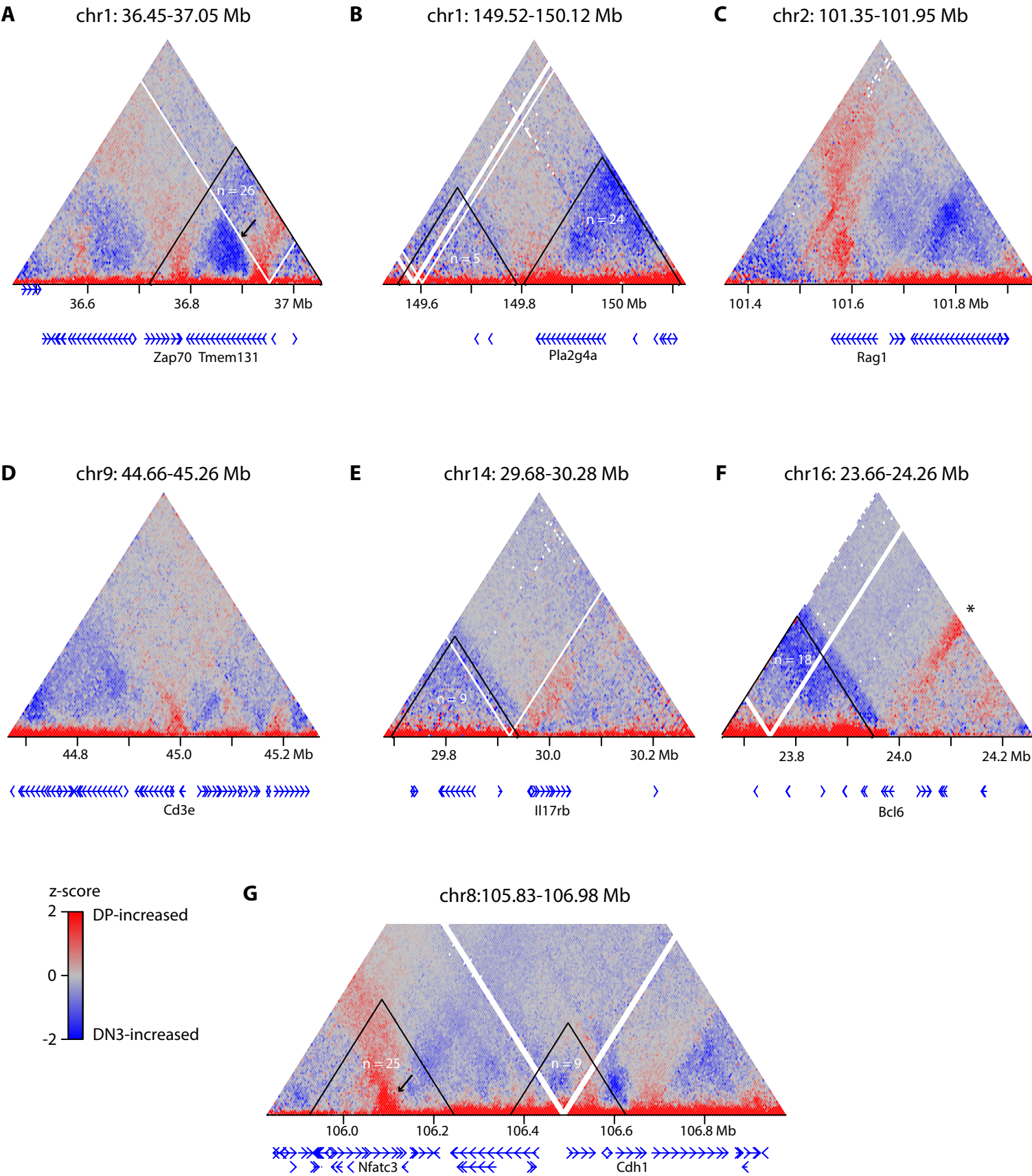

Supplement: S12 Fig — Differential Capture Hi-C maps between DP (enriched interactions in red) and DN3 (enriched interactions in blue) are shown for the captured regions: (A) Zap70; (B) Pla2g4a; (C) Rag1; (D) Cd3e; (E) Il17rb; (F) Bcl6; (G) Nfatc3/Cdh1. The black triangles denote the regions called as structurally different between the 2 cell types by CHESS, including the cell type–specific spatial domains around the Tmem131 and Nfatc3 genes (denoted by arrows). The number of sliding windows identified as structurally different is denoted in white text at each region. The widened domain border at Bcl6, denoted with an asterisk, was not identified by CHESS. Source data available in S1 Data. CHESS, Comparison of Hi-C Experiments using Structural Similarity; DN3, double negative; DP, double positive. (PDF) [file pbio.3002424.s012.pdf]
