# Supplementary material for: Transcription induces context-dependent remodeling of chromatin architecture during differentiation
Source: PLoS Biol. 2023 Dec 4;21(12):e3002424. doi: 10.1371/journal.pbio.3002424 (PMC10721200; doi:10.1371/journal.pbio.3002424)
Supplement: S1 Table — Overview of the genomic regions for which Capture Hi-C probes were designed, identified by the name of the gene over which they were centered. Red genes are more highly expressed in DP cells. Blue genes are more highly expressed in DN3 cells. Black genes are expressed equivalently in both thymocyte populations. Two captured regions were fused as one larger region (identified by *). (DOCX) [file pbio.3002424.s013.docx]

**Supplementary Tables**

S1 Table. Capture Hi-C strategy. Overview of the genomic regions for which Capture Hi-C probes were designed, identified by the name of the gene over which they were centered. Red genes are more highly expressed in DP cells. Blue genes are more highly expressed in DN3 cells. Black genes are expressed equivalently in both thymocyte populations. Two captured regions were fused as one larger region (identified by *).

| **Gene** | **Number of probes** | **Captured region (mm10)** |
| --- | --- | --- |
| *Bcl6* | 1725 | chr16:23,659,574-24,258,391 |
| *Nfatc3* | 3598* | chr8:105,833,089-106,979,086* |
| *Rag1* | 1639 | chr2:101,357,817-101,956,585 |
| *Cdh1* | 3598* | chr8:105,833,089-106,979,086* |
| *Il17rb* | 1726 | chr14:29,680,447-30,279,827 |
| *Pla2g4a* | 1578 | chr1:149,525,011-150,124,634 |
| *Cd3e* | 1925 | chr9:44,666,642-45,265,845 |
| *Zap70* | 1666 | chr1:36,456,898-37,055,359 |
