# Supplementary material for: Transcription induces context-dependent remodeling of chromatin architecture during differentiation
Source: PLoS Biol. 2023 Dec 4;21(12):e3002424. doi: 10.1371/journal.pbio.3002424 (PMC10721200; doi:10.1371/journal.pbio.3002424)
Supplement: S2 Table — Normalized RNA-seq expression values are given for the genes described in this study in ESCs, DN3, and DP cells, along with their ratios to highlight differential expression. (DOCX) [file pbio.3002424.s014.docx]

S2 Table. Cell type-specific expression of genes in this study. Normalized RNA-seq expression values are given for the genes described in this study in ESCs, DN3 and DP cells, along with their ratios to highlight differential expression.

| Gene | ESC | DN3 | DP | DP/DN | DN/DP | DN/ES | DP/ES |
| --- | --- | --- | --- | --- | --- | --- | --- |
| *Bcl6* | 10 | 2 | 468 | 213 |  |  | 47 |
| *Nfatc3* | 321 | 178 | 1643 | 9 |  |  | 5 |
| *Rag1* | 1 | 488 | 5995 | 12 |  | 610 | 7494 |
| *Cdh1* | 1905 | 124 | 3 |  | 41 |  |  |
| *Il17rb* | 2 | 265 | 75 |  | 4 | 139 | 39 |
| *Pla2g4a* | 7 | 92 | 4 |  | 23 | 14 |  |
| *Cd3e* | 1 | 762 | 1360 | 1.8 |  | 918 | 1639 |
| *Zap70* | 2 | 324 | 853 | 2.6 |  | 216 | 569 |
| *Tmem131* | 327 | 562 | 227 |  | 2.5 |  |  |
