# Supplementary material for: Transcription induces context-dependent remodeling of chromatin architecture during differentiation
Source: PLoS Biol. 2023 Dec 4;21(12):e3002424. doi: 10.1371/journal.pbio.3002424 (PMC10721200; doi:10.1371/journal.pbio.3002424)
Supplement: S3 Table — (DOCX) [file pbio.3002424.s015.docx]

S3 Table. Overview of Hi-C and Capture Hi-C datasets presented in this study.

| **Experiment** | **Number of reads** |
| --- | --- |
| DP Capture Hi-C, replicate 1 | 401,830,479 |
| DP Capture Hi-C, replicate 2 | 360,972,479 |
| DP *Dpn*II Hi-C, replicate 1 | 148,715,287 |
| DP *Dpn*II Hi-C, replicate 2 | 360,442,822 |
| DP *Hind*III Hi-C, replicate 1 | 176,274,361 |
| DP *Hind*III Hi-C, replicate 2 | 111,349,602 |
| DN3 Capture Hi-C, replicate 1 | 250,151,129 |
| DN3 Capture Hi-C, replicate 2 | 344,697,448 |
| DN3 *Dpn*II Hi-C, replicate 1 | 160,220,248 |
| DN3 *Dpn*II Hi-C, replicate 2 | 350,883,117 |
| DN3 *Hind*III Hi-C, replicate 1 | 140,836,381 |
| DN3 *Hind*III Hi-C, replicate 2 | 113,674,483 |
| ESC Capture Hi-C, replicate 1 | 342,156,642 |
| ESC Capture Hi-C, replicate 2 | 685,369,931 |
| ESC (CRISPRa *Bcl6*) Capture Hi-C, replicate 1 | 306,192,847 |
| ESC (CRISPRa *Bcl6*) Capture Hi-C, replicate 2 | 304,987,779 |
| ESC ΔCTCF (*Bcl6* promoter) Capture Hi-C, replicate 1 | 328,152,115 |
| ES ΔCTCF (*Bcl6* promoter) Capture Hi-C, replicate 2 | 360,340,711 |
| ESC (CRISPRa *Nfatc3*) Capture Hi-C, replicate 1 | 307,926,726 |
| ESC (CRISPR *Nfatc3*) Capture Hi-C, replicate 2 | 321,593,294 |
| ESC (CRISPRa *Il17rb*) Capture Hi-C, replicate 1 | 424,354,551 |
| ESC (CRISPRa *Il17rb*) Capture Hi-C, replicate 2 | 441,701,619 |
