# Supplementary material for: Transcription induces context-dependent remodeling of chromatin architecture during differentiation
Source: PLoS Biol. 2023 Dec 4;21(12):e3002424. doi: 10.1371/journal.pbio.3002424 (PMC10721200; doi:10.1371/journal.pbio.3002424)
Supplement: S4 Table — Spearman correlation coefficients between all biological replicates performed in this study. (DOCX) [file pbio.3002424.s016.docx]

**S4 Table. Reproducibility of Capture Hi-C.** Spearman correlation coefficients between all biological replicates performed in this study.

| **Experiment** | **Spearman correlation coefficient** |
| --- | --- |
| DN3 | 0.96 |
| DP | 0.97 |
| ESC | 0.97 |
| ESC (CRISPRa *Bcl6*) | 0.96 |
| ESC ΔCTCF (*Bcl6* promoter) | 0.96 |
| ESC (CRISPRa *Nfatc3*) | 0.95 |
| ESC (CRISPRa *Il17rb*) | 0.91 |
