# Supplementary material for: Transcription induces context-dependent remodeling of chromatin architecture during differentiation
Source: PLoS Biol. 2023 Dec 4;21(12):e3002424. doi: 10.1371/journal.pbio.3002424 (PMC10721200; doi:10.1371/journal.pbio.3002424)
Supplement: S5 Table — For pairwise combinations of the insulation scores, computed over different windows, their similarities are quantified with Spearman correlation coefficient, as well as their difference in distribution, as indicated by the p-values after two-tailed Kolgorov–Smirnov tests (KS p-val). (DOCX) [file pbio.3002424.s017.docx]

**S5 Table.** **Topological insulation similarities across cell type.** For pairwise combinations of the insulation scores, computed over different windows, their similarities are quantified with Spearman correlation coefficient, as well as their difference in distribution, as indicated by the p-values after two-tailed Kolgorov-Smirnov tests (KS p-val).

| **Window** | **DN3 vs ESC** | | **DP vs ESC** | | **DN3 vs DP** | |
| --- | --- | --- | --- | --- | --- | --- |
|  | **SCC** | **KS p-val** | **SCC** | **KS p-val** | **SCC** | **KS p-val** |
| 25 kb | 0.85 | 2.1x10^-11^ | 0.71 | 1.7x10^-8^ | 0.91 | 0.027 |
| 35 kb | 0.87 | 9.0x10^-12^ | 0.74 | 8.2x10^-6^ | 0.91 | 0.079 |
| 50 kb | 0.87 | 5.6x10^-11^ | 0.76 | 9.4x10^-5^ | 0.90 | 0.012 |
| 75 kb | 0.85 | 1.4x10^-5^ | 0.73 | 0.0041 | 0.91 | 0.12 |
