# Supplementary material for: Transcription induces context-dependent remodeling of chromatin architecture during differentiation
Source: PLoS Biol. 2023 Dec 4;21(12):e3002424. doi: 10.1371/journal.pbio.3002424 (PMC10721200; doi:10.1371/journal.pbio.3002424)
Supplement: S6 Table — As S4 Table, but on the lower-resolution genome-wide Hi-C data. SCC values are given separately for the region targeted by Capture Hi-C and for the whole genome (“all”). (DOCX) [file pbio.3002424.s018.docx]

**S6 Table.** **Topological insulation similarities genome-wide across cell type.** As **S4 Table**, but on the lower-resolution genome-wide Hi-C data. SCC values are given separately for the region targeted by Capture Hi-C and for the whole genome (“all”).

| **Window** | **DN3 vs ESC** | | | **DP vs ESC** | | | **DN3 vs DP** | | |
| --- | --- | --- | --- | --- | --- | --- | --- | --- | --- |
|  | ***Capture region*** | | ***All*** | ***Capture region*** | | ***All*** | ***Capture region*** | | ***All*** |
|  | ***SCC*** | ***KS pval*** | ***SCC*** | ***SCC*** | ***KS pval*** | ***SCC*** | ***SCC*** | ***KS pval*** | ***SCC*** |
| 70 kb | 0.72 | 3.1x10^-15^ | 0.49 | 0.46 | 2x10^-9^ | 0.39 | 0.78 | 0.03 | 0.61 |
| 100 kb | 0.74 | <2.2x10^-16^ | 0.55 | 0.49 | 5.6x10^-16^ | 0.43 | 0.83 | 0.30 | 0.73 |
| 150 kb | 0.74 | <2.2x10^-16^ | 0.58 | 0.50 | <2.2x10^-16^ | 0.45 | 0.84 | 0.20 | 0.78 |
