# Supplementary material for: Transcription induces context-dependent remodeling of chromatin architecture during differentiation
Source: PLoS Biol. 2023 Dec 4;21(12):e3002424. doi: 10.1371/journal.pbio.3002424 (PMC10721200; doi:10.1371/journal.pbio.3002424)
Supplement: S9 Table — (DOCX) [file pbio.3002424.s021.docx]

**S9 Table.** **Sequences of gRNAs used in this study.**

| **Target** | **gRNA sequence** |
| --- | --- |
| *Bcl6* promoter (CRISPRa) | 5’-AGGGGAGGACTCGGTGGCAG-3’ |
|  | 5’-CCCGAGGCATTCTGCCGGCC-3’ |
|  | 5’-TGTTCCGGGCGGCGGTGCTG-3’ |
|  | 5’-CGTGACGGCGGCGGAGCGGG-3’ |
| *Nfatc3* promoter (CRISPRa) | 5’-ATCGGGCGGAGCTCATGTCG-3’ |
|  | 5’-AGTCTCCAATTGGCCTACGT-3’ |
|  | 5’-TATCGCGTGAGTCCTCTGCG-3’ |
|  | 5’-CCAAGTTACGCCATCGAAGT-3’ |
| *Il17rb* promoter (CRISPRa) | 5’- TGGGCCAGGACGCGAAGGAC-3’ |
|  | 5’- GTGCCGCGAGCTGCTCCGTC-3’ |
|  | 5’- TCGACACGCCCCGCGCCTGC-3’ |
|  | 5’- TTGTCGCCCTCGGGGCTCGC-3’ |
| *Bcl6* CTCF site (deletion) | 5’-GTGTAGAAGGCGATGCTAAC-3’ |
|  | 5’-TCACCGTTAATTCATACCGA-3’ |
