# Supplementary material for: Transcription induces context-dependent remodeling of chromatin architecture during differentiation
Source: PLoS Biol. 2023 Dec 4;21(12):e3002424. doi: 10.1371/journal.pbio.3002424 (PMC10721200; doi:10.1371/journal.pbio.3002424)
Supplement: S10 Table — (DOCX) [file pbio.3002424.s022.docx]

**S10 Table.** **Sequences of qRT-PCR primers used in this study.**

| **Gene** | **Sequence** |
| --- | --- |
| *Gapdh* | 5’-CATCACTGCCACCCCAGAAGACTG-3’ |
|  | 5’-ATGCCAGTGAGCTTCCCGTTCAG-3’ |
| *Bcl6* | 5’-cacgcggtattgcacctt-3’ |
|  | 5’-catccacacaggagagaaacc-3’ |
| *Nfatc3* | 5’-CACCATCATTTCAGCTCCAA-3’ |
|  | 5’-GCACTCAAAGGGTTTAGGAC-3’ |
| *Il17rb* | 5’-gacaacagacgcatgctggg-3’ |
|  | 5’-gtgctccttccttgcctccaag-3’ |
