# Supplementary material for: Transcription induces context-dependent remodeling of chromatin architecture during differentiation
Source: PLoS Biol. 2023 Dec 4;21(12):e3002424. doi: 10.1371/journal.pbio.3002424 (PMC10721200; doi:10.1371/journal.pbio.3002424)
Supplement: S12 Table — Red sequence denotes Illumina sequencing adapters. (DOCX) [file pbio.3002424.s024.docx]

**S12 Table.** **Sequences of 4C-seq primers used in this study.** Red sequence denotes Illumina sequencing adapters.

| **Name** | **Sequence** |
| --- | --- |
| *Bcl6* reading primer | 5’-AATGATACGGCGACCACCGAGATCTACACTCTTTCCCTACACGACGCTCTTCCGATCTCTTAAGGAGCCCACAGGAGTG-3’ |
| *Bcl6* non-reading primer | 5’-CAAGCAGAAGACGGCATACGAGCTCTTCCGATCTGGGAGTCAAGGGATAAGACACA-3’ |
| *Rag1* reading primer | 5’-AATGATACGGCGACCACCGAGATCTACACTCTTTCCCTACACGACGCTCTTCCGATCTAGGGACAAAACTTCTATTCATGATC-3’ |
| *Rag1* non-reading primer | 5’-CAAGCAGAAGACGGCATACGAGCTCTTCCGATCTGGTCTCTCCCTTATATTCTTATCCTAA-3’ |
