# Supplementary material for: Transcription induces context-dependent remodeling of chromatin architecture during differentiation
Source: PLoS Biol. 2023 Dec 4;21(12):e3002424. doi: 10.1371/journal.pbio.3002424 (PMC10721200; doi:10.1371/journal.pbio.3002424)
Supplement: S13 Table — (DOCX) [file pbio.3002424.s025.docx]

**S13 Table.** **Previously published GEO datasets used in this study.**

| **Dataset** | **GEO accession number** | **Reference** |
| --- | --- | --- |
| ESC RNA-seq | GSE29278 | (1) |
| ESC H3K27ac ChIP-seq | GSE29218 | (1) |
| ESC CTCF ChIP-seq | GSE49847 | (2) |
| DN3 RNA-seq | GSE109125 | (3) |
| DN3 H3K27ac ChIP-seq | GSE80138 | (4) |
| DN3 CTCF ChIP-seq | GSE41743 | (5) |
| DN3 RNA polymerase II ChIP-seq | GSE55635 | (6) |
| DN3 H3K4me1 ChIP-seq | GSE56395 | (7) |
| DN3 H3K27me3 ChIP-seq | GSE61149 | (8) |
| DP RNA-seq | GSE109125 | (3) |
| DP H3K27ac ChIP-seq | GSE63732 | (9) |
| DP CTCF ChIP-seq | GSE41743 | (5) |
| DP RNA polymerase II ChIP-seq | GSE29362 | (10) |
| DP H3K4me1 ChIP-seq | GSE20898 | (11) |
| DP H3K27me3 ChIP-seq | GSE61149 | (8) |

**References**

1. Y. Shen, F. Yue, D.F. McCleary, Z. Ye, L. Edsall, S. Kuan, U. Wagner, J. Dixon, L. Lee, V.V. Lobanenkov, B. Ren, A map of the cis-regulatory sequences in the mouse genome. Nature 488, 116-20 (2012). 10.1038/nature11243.
2. F. Yue, Y. Cheng, A. Breschi, J. Vierstra, W. Wu, T. Ryba, R. Sandstrom, Z. Ma, C. Davis, B.D. Pope, et al. A comparative encyclopedia of DNA elements in the mouse genome. *Nature* **515**, 355-364 (2014). 10.1038/nature13992.
3. H. Yoshida, C.A. Lareau, R.N. Ramirez, S.A. Rose, B. Maier, A. Wroblewska, F. Desland, A. Chudnovskiy, A. Mortha, C. Dominguez, J. Tellier, E. Kim, D. Dwyer, S. Shinton, T. Nabekura, Y. Qi, B. Yu, M. Robinette, K.W. Kim, A. Wagers, A. Rhoads, S.L. Nutt, B.D. Brown, S. Mostafavi, J.D. Buenrostro, C. Benoist, Immunological Genome Project, The cis-regulatory atlas of the mouse immune system. *Cell* **176**, 897-912.e20 (2019). 10.1016/j.cell.2018.12.036.
4. S. Klein-Hessling, R. Rudolf, K. Muhammad, K.P. Knobeloch, M.A. Maqbool, P. Cauchy, J.C. Andrau, A. Avots, C. Talora, V. Ellenrieder, I. Screpanti, E. Serfling, A.K. Patra, A threshold level of NFATc1 activity facilitates thymocyte differentiation and opposes notch-driven leukemia development. *Nat. Commun*. **7**, 11841 (2016). 10.1038/ncomms11841.
5. H.Y. Shih, J. Verma-Gaur, A. Torkamani, A.J. Feeney, N. Galjart, M.S. Krangel, Tcra gene recombination is supported by a Tcra enhancer- and CTCF-dependent chromatin hub. *Proc. Natl. Acad. Sci. USA*. **109**, E3493-3502 (2012). 10.1073/pnas.1214131109.
6. A. Pękowska, T. Benoukraf, J. Zacarias-Cabeza, M. Belhocine, F. Koch, H. Holota, J. Imbert, J.C. Andrau, P. Ferrier, S. Spicuglia, H3K4 tri-methylation provides an epigenetic signature of active enhancers. *EMBO J*. **30**, 4198-4210 (2011). 10.1038/emboj.2011.295.
7. P. Cauchy, M.A. Maqbool, J. Zacarias-Cabeza, L. Vanhille, F. Koch, R. Fenouil, M. Gut, I. Gut, M.A. Santana, A. Griffon, J. Imbert, C. Moraes-Cabé, J.C. Bories, P. Ferrier, S. Spicuglia, J.C. Andrau, Dynamic recruitment of Ets1 to both nucleosome-occupied and -depleted enhancer regions mediates a transcriptional program switch during early T-cell differentiation. *Nucleic Acids Res*. **44**, 3567-85 (2016). 10.1093/nar/gkv1475.
8. A. Oravecz, A. Apostolov, K. Polak, B. Jost, S. Le Gras, S. Chan, P. Kastner, Ikaros mediates gene silencing in T cells through Polycomb repressive complex 2. *Nat. Commun.* **6**, 8823 (2015). 10.1038/ncomms9823.
9. L. Vanhille, A. Griffon, M.A. Maqbool, J. Zacarias-Cabeza, L.T.M. Dao, N. Fernandez, B. Ballester, J.C. Andrau, S. Spicuglia, High-throughput and quantitative assessment of enhancer activity in mammals by CapStarr-seq. *Nat. Commun*. **6**, 6905 (2015). 10.1038/ncomms7905.
10. F. Koch, R. Fenouil, M. Gut, P. Cauchy, T.K. Albert, J. Zacarias-Cabeza, S. Spicuglia, A.L. de la Chapelle, M. Heidemann, C. Hintermair, D. Eick, I. Gut, P. Ferrier, J.C. Andrau, Transcription initiation platforms and GTF recruitment at tissue-specific enhancers and promoters. *Nat. Struct. Mol. Biol*. **18**, 956-963 (2011). 10.1038/nsmb.2085.
11. G. Wei, B.J. Abraham, R. Yagi, R. Jothi, K. Cui, S. Sharma, L. Narlikar, D.L. Northrup, Q. Tang, W.E. Paul, J. Zhu, K. Zhao, Genome-wide analyses of transcription factor GATA3-mediated gene regulation in distinct T cell types. *Immunity* **35**, 299-311 (2011). 10.1016/j.immuni.2011.08.007.
