# Supplementary material for: Transcription induces context-dependent remodeling of chromatin architecture during differentiation
Source: PLoS Biol. 2023 Dec 4;21(12):e3002424. doi: 10.1371/journal.pbio.3002424 (PMC10721200; doi:10.1371/journal.pbio.3002424)
Supplement: Attachment — Submitted filename: Chahar_ResponseToReviewers.docx [file pbio.3002424.s027.docx]

**Point-by-Point Response to Prior Peer Review**

**Reviewer 1**

*3D genome organization, and particularly Topologically Associating Domains, can guide cell type-specific transcriptional programs. This involves the reorganization of genome architecture during cellular differentiation, with increasingly more players identified to be involved in this process. The diversity of this reorganization, and particularly how various players engage with each other at individual loci, remains largely uncharacterized though. Here, the authors used ultra-high-resolution Capture Hi-C in three linked cell types (pluripotent embryonic stem cells, early-stage double negative 3 thymocytes, and later stage double positive thymocytes) at eight genomic domains that contain both TAD boundaries and differentially expressed genes. First, confirming and expanding upon previous observations, they show that genome organization becomes increasingly defined upon cellular differentiation, although with marked differences at individual TAD boundaries (Fig. 1,2). Next, by focusing on individual gene loci, they make interesting observations about the diversity of genome reorganization that can accompany transcriptional changes. In a first example (Fig. 3), they show how PolII pause release appears to 'widen' a boundary between TADs. This unique observation constitutes a new addition to the toolkit of 3D chromatin remodeling. Yet, CRISPRa experiments in a non-expressing cell type were not able to reproduce this observation, suggesting that this widening of boundaries incorporates other mechanisms as well. Indeed, they identify promoter-enhancer contacts as a possible additional player. In a second set of comparisons (Fig. 4), they convincingly show that active transcription can create sub-domains within TADs that precisely match the gene body. Until now, such structures had not been reported in mammalian cells, thereby identifying a further involvement of PolII in genome organization.*

*This study, using the capacity of Capture Hi-C to generate 3D contact maps at extremely high coverage and resolution (2 kb), reveals a number of unique reorganization events that have gone unnoticed both in averaged genome-wide studies and in studies at the limited number of model loci that are most frequently investigated. As such, this experimental design confirms the importance of giving individual attention to a range of different gene loci, rather than focusing on the population average or a limited number of model loci.*

We thank the reviewer for their positive comments and concise summary of the impact of our work.

*At the same time, this is also a major limitation of the study: it encompasses eight domains, which can be further divided into loci with increased, reduced, or unchanged gene activity. The identified mechanisms remain generally limited to one or two loci, therefore leaving open if such mechanisms are more commonly used or if they are coincidental outliers. In conclusion, this study reports a number of interesting additions to the collection of 3D genome reorganization events during transcriptional activation. Additional studies, incorporating both larger numbers of genomic loci and different differentiation contexts, will be required to establish the prevalence of such reorganization events.*

We agree with the reviewer, but find such systematic high-resolution studies to be beyond the scope of this article. We have now bolstered our work with demonstration of TAD remodeling on induction of a second gene (*Il17rb*). One of the important findings of this study already is that chromatin architectures clearly have context-dependent sensitivities to developmental/transcriptional-coupled remodeling, and the differences in how *Il17rb* or *Nfatc3* local chromatin architecture is altered (whether a new border is created or not, respectively) on gene induction is just as important a finding as their similarities (a new spatial domain created in both).

*Overall, I find the experiments in this study of high quality and I have no major suggestions for additional experiments or analyses. In contrast, the presentation of the results is not always the easiest (text and figures), and could benefit from restructuration.*

*1. The text, and particularly the introduction, contains many complex and conditional sentences that often convey multiple messages. The information will be easier to access if the authors can split or reorganize sentences to focus on a single message.*

The text has been edited to reduce the size of some of the longer sentences. We also note that Reviewer 3 (see below) actually praised the clarity of the manuscript.

*2. The information about individual gene loci is often spread out over multiple panels in the main and supplemental information, complicating the interpretation. It would be helpful if. One such example is figure 3, where the actual Hi-C maps are in Fig. S5, a differential Hi-C matrix is in Fig. 3A (together with PolII tracks), the CTCF binding is in Fig. 3B and H3K27ac tracks are in Fig. 3D. As the number of figures is not limiting, I would propose to regroup the most relevant data in single panels.*

*a. For each gene of focus, indicate its activity status for the cell type in the figure.*

*b. Add actual Hi-C maps to the main figure, instead of only differential matrixes.*

*c. Group, where possible, all genomic tracks together in a single panel. Ideally, this includes systematically including CTCF and RNA-seq tracks as well.*

These changes have all now been incorporated in extensively reworked figures. As opposed to one very large supplemental figure showing all of the captured regions, we have now generated a separate supplemental figure for each region, incorporating fuller epigenomic profiles. The cell type specificity of the relevant genes is now explicitly annotated. Each differential Hi-C map is now shown directly alongside the original Hi-C maps used in the comparisons.

*Specific comments:*

*- Line 143: Figure S2 is discussed before S1B-D. Better to split up figure S1?*

With the new figure setup, they are all now presented in the order in which they are first described.

*- Line 219: I don't understand how the authors conclude the boundary becomes leakier. Is this based on the blue Z-score in the left TAD? Signal intensities between the 2 TADs in Fig. S5A, if anything, appear reduced in DP cells. Instead, the reduced insulation score over this entire TAD in DP cells suggests the TAD itself becomes less well organized.*

We apologize for the confusion caused; the reviewer is absolutely right. We have now reworded the relevant section to talk only about the border becoming broader, not leakier.

*- Line 234: although CTCF binding is conserved within the widened boundary, it appears that peak levels towards the TTS in DP cells become increased. Clustering of CTCF binding has been linked to broader "zones of transition" between TADs (https://www.ncbi.nlm.nih.gov/pubmed/31887284). Should these dynamics be taken into consideration for this boundary as well?*

The relevant citation has been added and we have mentioned this possibility.

*- Line 238: the CRISPRa experiments would have been more relevant in the DN3 cells, where PolII is strongly paused and the promoter-enhancer loop is in place. Why did the authors use the stem cell system?*

Unfortunately, this is technically unfeasible, because *ex vivo* thymocytes cannot be cultured for the required times. We originally planned to do such experiments in the thymocyte-like cell line, P5424, but promoter Capture Hi-C experiments within our group (unpublished) showed that their chromatin architecture does not really resemble DN3 or DP cells. We were thus restricted to ESCs, which were suitable to show transcription-driven creation of new domains (*Nfatc3* and *Il17rb*), but indeed do not recapitulate the stalled polymerase and enhancer interaction at the *Bcl6* locus.

*- Line 240: The impact of PolII on chromatin organization will likely depend on the absolute level of transcription. Do the authors know how their CRISPRa activation compares to the activity in DP cells?*

We have now included a new supplemental table (**S2 Table**), showing the fold-changes in expression of the relevant genes between ESC, DN3 and DP cells, as determined by RNA-seq studies. For *Nfatc3*, the fold-inductions within ESC, as determined by RT-qPCR, are within the same sort of range as the thymocyte transition inductions. For *Il17rb*, the total induced gene expression levels are comparable to induced *Nfatc3*, but this actually represents a large overexpression compared to thymocytes. This information is included in the revised text.

*- Line 274: the interaction domains as described here resemble contact domains, as first described in https://www.ncbi.nlm.nih.gov/pubmed/25497547. These include H3K36me3-marked domains, which are directly linked to transcriptional elongation. Could these phenomena be linked?*

Indeed they could, since RNA polymerase II and H3K36me3 “domains” are highly correlated anyway. In response to Reviewer 2’s comments (see below), we have now extended the genome-wide meta-analysis of the thymocyte Hi-C data to include other histone marks, including H3K36me3. As expected from the results with RNA polymerase II from the previous version of the manuscript, there is a positive correlation between intra-gene compaction and median H3K36me3 signal, although this is rather weak for DP thymocytes (we believe due to lower quality of this particular ChIP-seq dataset). Because of the confusion this might cause, we have omitted this particular histone mark from the results in **Fig 5**.

*- Section 336: the authors raise an important question here, with a further reason why subtle changes have been overlooked is that most analyses look for genome-wide averages. The authors mention the CHESS tool to identify more subtle changes, which has been successfully used on Capture Hi-C data as well. This raises the question of why the authors did not use this tool themselves.*

This is an excellent suggestion and we have now included CHESS analysis (**S12 Fig**). Whereas it did succeed in calling the DN3- or DP-specific spatial domains, the broadened border at *Bcl6* was not picked up, and there were several other “false positives” called as structurally similar, even though close inspection of the Capture Hi-C (and differential) maps showed no apparent difference apart from the systematic tendency for DP to have stronger on-diagonal interactions, in line with its generally increased chromatin compaction. When we applied CHESS to the Hi-C maps, trialing different parameters, none of the called “differences” made sense on visual inspection, and previously identified thymocyte-specific spatial domains (compared to ESCs; e.g. *Runx1*, **S9C Fig**) were not called. We have modified the discussion accordingly to say that there is still scope for other analytical methods, beyond insulation scores *and* CHESS.

*- Fig. 1B and S1D: the dashed lines to indicate interactions partially obscure the interesting features (dots, stripes). Could the authors use arrows instead?*

We have now made this change.

**Reviewer 2**

*A case study of how HiC data capture the changes in topology upon differentiation of thymocytes. The authors provide a very honest description of the detected changes in insulation scores and additional changes at sub-TAD levels. It is of interest that some of the main changes may occur upon induction of transcription. The authors propose to tackle the question of TAD remodeling upon cell differentiation into mouse thymocytes. They score insulation in three stages and identify some candidates where insulation may vary yet with no clear systematic rule to account for such changes. They focus on individual cases resembling compartmentalized domains reported in Drosophila where the whole gene unit appears to define preferential interactions upon activation of the gene in thymocytes. The authors suggest that more changes may occur at sub-TADs levels in a transcription-coupled fashion. They provide some support for their observations by showing how differentiation may reinforce a global correlation between RNA polymerase II levels and Hi-C contacts.*

*The study is very honest in stating a number of limits of the analyses performed and of the conclusions that can be (or cannot be) drawn. The work has some interest with respect to finding net changes in a sub-TAD structure, upon induction of a thymocyte-specific gene, which is not necessarily related to CTCF insulation.*

We thank the reviewer for their interest.

*It is not totally clear how relevant the scoring of TAD borders was given the low number of borders scored.*

*Since the authors use a binary yes/no approach in identifying borders, a complementary quantitative analysis would be required to validate that the borders lost in one cell type are truly lost instead of just decreasing.*

In the manuscript, we have tried to emphasize the greater relevance in studying global distributions of insulation scores, their correlations and principal component analysis (**Figs 2A-C**) to get overall ideas of architectural changes. These are indeed quantitative, as the reviewer asked. We have also been honest in saying that our TAD border calling is directed at only calling the most robust ones. Some cases, such as the thymocyte-specific domain around the *Cd3* genes, are detected by this “conventional” analysis (**Fig 2D-F**). However, we highlight in the Discussion that the most pertinent developmental chromatin architecture changes (*Bcl6, Nfatc3*, *Tmem131* and *Il17rb*; **Figs 3-4**; **S11 Fig**) we observed are NOT accompanied by changes in TAD borders as called from simple insulation analysis. We therefore do not believe we have placed undue emphasis on the TAD border calling, and have only used the analysis for completeness.

*Also, the authors acknowledge the lower resolution of the HiC data used in this study. When using capture Hi-C, it follows that such analysis would be biased towards identifying borders at promoters. The authors need to clarify this and draw conclusions accordingly.*

We believe the reviewer has misunderstood our Capture Hi-C design. We have not designed probes to promoters, like for most published (Promoter Capture Hi-C) datasets, but have designed tiled probes to cover all sufficiently long restriction fragments within entire ~600 kb regions. Variation in probe capture efficiency, just like variation in digestion efficiency during the Hi-C process, is accounted for by the matrix balancing normalization as for conventional Hi-C analysis. In any case, there is no reason to expect a bias for promoters in this strategy. If the reviewer means instead that we are biased in looking at architectures around chosen thymocyte genes, this was indeed the goal of the study. As stated in the Introduction and Results sections, we purposefully chose regions of the genome where architectural changes were most likely to be observed if there was a real link to underlying gene expression.

*A number of interesting observations are reported such as the specific sub-TADs near genes "specifically expressed in thymocytes". Yet it is not what the data show (Fig. 2D). The data show one case, but not a comparison allowing the reader to assess whether there is any specificity in setting up a sub-TAD, particularly at genes that are induced in thymocyte. This needs further quantification.*

We apologize for this part of the manuscript not being clear. We do not wish to claim that these domains are in any way specific to thymocyte-specific genes, just that we observed such a phenomenon at *Cd3* (with the Capture Hi-C) and *Runx1* (with conventional Hi-C), which just happened to fit their thymocyte-specific expression AND specific CTCF recruitment. We have reworded the text accordingly to avoid the confusion.

*The possibility that transcription plays a role in setting up TADs or borders was already suggested.*

We agree, and this comprises a large part of the literature overview within our Introduction, particularly the apparent controversy. We note that this manuscript is, to our knowledge, the first to show a direct remodeling of a spatial chromatin domain via transcriptional induction.

*The scoring of intra-gene contacts as a function of RNA Polymerase II is of interest though the relative quantification of HiC is difficult. Such analysis would deserve some better quantification possibly comparing the correlations depending on multiple ChIP-seq datasets. Furthermore, it may be of interest to do the same analysis as a function of pausing status.*

We do not understand from the reviewer’s comments how we could make the quantification of the Hi-C data any better. We extended the analysis to other epigenomic features where datasets exist for DN3 and DP thymocytes, a subset of which is now included in **Fig 5**. The table below gives the Spearman correlation coefficients between intra-gene contact strength and the median gene signal for the different features we assessed. The pausing index for each gene was determined as the summed RNA polymerase II ChIP-seq signal at the promoter and TSS (-500 to +500 relative to the TSS), divided by the summed RNA polymerase II ChIP-signal over the rest of the gene body (normalized per kb to account for gene length differences).

| **Feature** | **DN3** | **DP** |
| --- | --- | --- |
| RNA polymerase II | 0.62 | 0.61 |
| RNA-seq | 0.61 | 0.60 |
| DNase | 0.61 | 0.64 |
| H3K4me1 | 0.70 | 0.70 |
| H3K27ac | 0.71 | 0.71 |
| H3K36me3 | 0.62 | 0.18 |
| H3K27me3 | -0.26 | -0.32 |
| Pausing index | 0.02 | 0.01 |

With the exception of DP H3K36me3 (which we believe to be a dataset of lower quality), there is a clear positive correlation between “active” histone marks and chromatin accessibility and inter-gene contacts, and a weaker but significant negative correlation with the repressive H3K27me3 mark. There is no correlation between pausing index and intra-gene contact strength, which may be expected from our results on *Bcl6*, which do not show a cell type difference in “domain strength”, but rather a widening of the TAD border. Since most of the active marks are known to correlate anyway, for a simpler figure we only included RNA polymerase II, H3K27ac, H3K4me1 and H3K27me3 in the final figure.

**Reviewer 3**

*In this manuscript, Chahar, Ben Zouari, and colleagues set out to decipher the mechanistic link between transcription and TAD organization. To this end, they performed Capture Hi-C for 8 regions in mES cells and at 2 stages of mouse thymocyte development (DN3 and DP). In addition, they performed perturbation experiments to identify the causal relationship between transcription and TAD formation.*

*Strengths of the manuscript:*

*- The data are solid and generally of high quality.*

*- The manuscript is well written and the data are clearly presented; it is, therefore, easy to follow and a pleasure to read.*

We thank the reviewer for their positive comments.

*Weakness of the manuscript:*

*- The authors focus on 8 regions: 3 that are upregulated on DN3-to-DP transition (Bcl6, Nfatc3, Rag1), 3 that are downregulated (Cdh1, Il17rb, Pla2g4a), and 2 that are unchanged (Cd3e, Zap70). It is in my opinion in principle fine to use a few model loci to arrive at general mechanisms, but in this case, the observations are not consistent across the loci. For example, one conclusion of the paper is that "sub-domains were formed around some activated genes without changes in CTCF binding". This seems to be true for Nfatc3 and Rag1 (and Tmem131, an additional gene present in the Zap70 locus, that appears to be upregulated in DN3 cells), but is not clear for Bcl6 (for which the authors describe "broadening of the TAD boundary" close to the promoter). More importantly, such a subdomain also appears/strengthens at the Cd3e and Il17rb loci. Since such a subdomain arises at 2 out of 3 upregulated but also at downregulated and unchanged genes, it is not clear that this is linked to transcription. Furthermore, ectopic induction of transcription recapitulates the changes during development at the Nfatc3 locus, but not at the Bcl6 locus. It, therefore, remains unclear to what extent the findings of the authors can be linked to changes in gene expression. I appreciate that the authors are careful to not overinterpret their data and draw any general conclusions, but I do think it would be of interest to perform a more complete analysis, taking additional regulatory mechanisms into consideration, to explain the observations.*

In the original version of the manuscript, *Il17rb* was indeed somewhat negatively cited as a counter-example to transcriptional remodeling of chromatin architecture, since downregulation of the gene on DN3-to-DP transition had negligible effects on spatial domains, compared to striking remodeling at *Tmem131*. However, a closer look at this locus shows that there are actually thymocyte-specific architectures, which are not present in ESCs where *Il17rb* is completely silent, raising the possibility that there are different thresholds for each locus to be remodeled. We have now recapitulated some features of this architectural remodeling on overexpression of the gene in ESCs (**Fig 4J-K**), bolstering our initial claim that transcription can directly remodel chromatin architecture.

As now described in the Discussion, the genome-wide meta-analyses plus our two direct experiments at *Nfatc3* and *Il17rb* strongly suggest that transcription indeed plays a role in organizing local spatial chromatin domains, but that there are clearly varying sensitivities of different loci to be remodeled. In addition, our results allude to other features that are likely to affect chromatin architecture, including differential CTCF binding (locus-specific sensitivity of TAD borders to numbers of CTCF sites deleted is well described in the literature; we reviewed this in *J Mol Biol* **432**, 653-64 (2020), for example) and promoter-enhancer interactions (suggested by our results with *Bcl6*, although our experimental setup was limited in being able to directly test this feature). We believe the Discussion now balances describing the importance of the direct transcriptional link to chromatin architecture, weighed against other mechanisms (some suggested here, others unknown) that affect locus sensitivity to remodeling.

*- The nomenclature of chromatin domains is somewhat confusing. Since TADs are generally defined as domains that are formed by loop extrusion (see e.g. Mirny & Dekker Cold Spring Harb. Perspect. Biol. 2021; Beagan & Phillips-Cremins Nat. Genet. 2020), some of the domains that do not align with CTCF sites are likely (small) compartments. A better definition of the different structures in the investigated regions might contribute to a clearer interpretation of some of the conflicting results.*

The original description of TADs actually predates any descriptions of loop extrusion. But since there is a lot of confusion around this, we have changed the term in the manuscript to “spatial chromatin domain” to describe domains of enriched Hi-C internal contacts and is a term which is agnostic to the molecular mechanism generating them.

*- The authors do not generally discuss the potential role of enhancers in remodeling chromatin architecture (except at the Bcl6 locus).*

In the general term, we have discussed this. In the specific term, only *Bcl6* has a relevant distal enhancer within our captured regions, and Hi-C needs to be at extremely high depth to identify promoter-enhancer contacts that are not CTCF-mediated loops (e.g. Bonev et al., 2017).

*Although the presented work contains a few interesting observations, the current data do not make a strong contribution to our general understanding of the mechanistic link between transcription and TAD organization.*

We disagree. To our knowledge, this is the first direct demonstration of transcriptionally mediated spatial domain remodeling, shown at two independent loci.

*- The figures generally show very little annotation (open chromatin, chromatin modifications, etc.), which makes the data sometimes a bit difficult to interpret.*

In reply to Reviewer 1’s comments, we have grouped together the Hi-C maps with their relevant epigenomic profiles, in order make these data easier to interpret. We could add extra profiles, but since most correlate with the ones already shown, we think this will actually make the figures more confusing, not less.

*- The authors stress the importance of using high-resolution approaches to identify tissue-specific changes in TADs. I agree that this is important, but conceptually not novel, and there are many studies that have used a similar approach and appear relevant but are not discussed (e.g. Andrey et al. Genome Res. 2017; Paliou et al. PNAS 2019; Oudelaar et al. Nat. Commun. 2022).*

The specific references mentioned by the reviewer are not directly relevant. Andrey et al. uses sparse probes (similar to Promoter Capture Hi-C), so is not equipped to analyze domain structures. Paliou et al. actually observed looping changes, and not spatial domain changes. Aljahani (Oudelaar) et al. looks at gross architectural changes brought about by cohesin or CTCF ablation, and does not address developmental changes. We have included many, more relevant, citations in the manuscript, and indeed have not claimed the need for high resolution to be a novel concept.
